# Efficient 1 µm Laser Emission of Czochralski-Grown Nd:LGSB Single Crystal

**DOI:** 10.3390/ma12122005

**Published:** 2019-06-22

**Authors:** Catalina-Alice Brandus, Stefania Hau, Alin Broasca, Madalin Greculeasa, Flavius-Marian Voicu, Cristina Gheorghe, Lucian Gheorghe, Traian Dascalu

**Affiliations:** 1 National Institute for Laser, Plasma and Radiation Physics, Laboratory of Solid-State Quantum Electronics, Atomistilor 409, Magurele 077125, Ilfov, Romania; stefania.hau@inflpr.ro (S.H.); alin.broasca@inflpr.ro (A.B.); madalin.greculeasa@inflpr.ro (M.G.); flavius.voicu@inflpr.ro (F.-M.V.); cristina.gheorghe@inflpr.ro (C.G.); traian.dascalu@inflpr.ro (T.D.); 2 Doctoral School of Physics, University of Bucharest, Faculty of Physics, Magurele 077125, Ilfov, Romania

**Keywords:** Nd-doped borate crystals, end pumping, passive Q-switching, SESAM mode-locking

## Abstract

A 5.0-at.% Nd-doped La_0.64_Gd_0.41_Sc_2.95_(BO_3_)_4_ (Nd:LGSB) borate laser crystal was successfully grown by the Czochralski method, for the first time to our knowledge. The spectroscopic properties of the grown crystal are discussed and 1 µm laser emission, under end-pumping with a fiber-coupled diode laser at 807 nm, is reported. A *c*-cut Nd:LGSB medium yielded 1.35 W continuous-wave output power at 0.63 overall optical-to-optical efficiency, with respect to the absorbed pump power, together with the high 0.68 slope efficiency. With an *a*-cut Nd:LGSB sample, 0.81 W output power at 0.52 optical-to-optical efficiency was obtained. The laser emission performances under quasi-continuous wave pumping are presented as well, for both *c*-cut and *a*-cut crystals. Passive Q-switching was investigated with a semiconductor saturable absorber mirror (SESAM). Laser pulses with 2.2 µJ energy and 32.8 ns durations were recorded from *a*-cut Nd:LGSB. The average output power reached 0.36 W at 1.55 W absorbed pump power. Passive mode-locking with SESAM was achieved in a long Z-type resonator. Ultrashort pulses with 0.19 W average power, 1.63 nJ energy, and 1.43 ps pulse duration, at 118 MHz repetition rate, are demonstrated for the *a*-cut Nd:LGSB medium.

## 1. Introduction

Lasers emitting around 1 μm are successfully employed in various fields, such as medicine, biology, industry, laser ignition for aeronautics and automotive, laser display, laser sensing, high resolution microscopy, spectroscopy, or research [1,2,3,4,5,6]. One-micron lasers are typically attained with Nd- or Yb-doped active media [7,8]. With its narrow emission bandwidth (full width at half maximum FWHM up to a few nm), the Nd trivalent ion is suitable in developing picosecond (ps) lasers. The Yb ion, on the other side, due to the larger emission bandwidth (tens of nm wide), is often used for the development of ultrashort pulsed laser sources with pulses of hundreds down to few tens of femtoseconds (fs) in duration. However, the Nd-doped media have a small lasing threshold for 1 μm radiation, this being a key advantage as compared to Yb-doped media emitting in the same wavelength range. 

Until now, different Nd-doped single crystals were discovered, among which the most famous are Nd:YAG, Nd:YVO_4_, and Nd:GdVO_4_. Some studies focused also on partially disordered crystals, such as Nd:CNGS, Nd:GGG, or Nd:ASL, due to their inhomogeneously broadened absorption and emission lines [9,10,11]. Usually, the Nd:YVO_4_ and Nd:YAG CW lasers pumped longitudinally with a laser diode at ~0.81 μm have shown slope efficiencies around 60%, with respect to the absorbed pump power (*P_abs_*), with the crystals used in the experiments being anti-reflection (AR) coated at the lasing wavelength [12,13,14,15]. For instance, with a 5 mm long, 1.0-at.% Nd:YAG crystal, a value of 60.6% of the slope efficiency was determined [15], while for Nd:YVO_4_ a slope efficiency of 66.6% was reported for a flat-flat resonator [16]. Improved slope efficiencies, up to 80% in Nd:YVO_4_, were obtained using different pumping geometries or a direct pumping approach, i.e., pumping at 880 or 914 nm [17,18,19,20,21,22].

Previously, Nd-doped lanthanum scandium borate (Nd:LSB) was investigated and laser emission was reported in the continuous wave (CW) operation mode, as well as in pulsed regime [23,24,25]. Ostroumov et al. achieved very good performances from a CW Nd:LSB laser. For 10.0-at.% Nd-doped LSB, they claimed output performances similar to those observed with 1.0-at.% Nd:YVO_4_ [26]. They have measured more than 500 mW output power at 1.06 μm, *P_abs_*~ 800 mW, for a plane-concave resonator with a 1% transmission of output coupler. Additionally, Romero et al. demonstrated CW operation in the case of a 10.0-at.% Yb:LSB with a slope efficiency of 64%, for a short crystal of 1.1 mm thickness, the maximum output power was around 500 mW for *P_abs_*~850 mW [27]. However, it is known that LSB medium crystallizes in a centrosymmetric monoclinic structure and, therefore, it cannot be used directly for frequency doubling. Thus, Ostroumov et al. reported efficient intra-cavity frequency doubling of the Nd:LSB laser, outputting 800 mW in the green range with 1 mm thick KTP, mounted on the surface of the LSB crystal, for 3 W incident pump power [26]. In 2016, Gheorghe et al. proposed a new crystal as a promising future nonlinear optical (NLO) material for frequency conversion applications, i.e., the lanthanum scandium gadolinium borate (LGSB). They demonstrated that the monoclinic structure of LSB can be converted to the trigonal structure (space group *R32*) over a certain level of Gd doping concentration. LGSB is isostructural to YAl_3_(BO_3_)_4_ (YAB) crystal and it has comparable NLO properties with those of YAB [28]. The advantage of the LGSB crystal is that it can be grown to a large size and with a high quality by the Czochralski method. In the same year, Khaled et al. achieved efficient laser emission from a Czochralski grown 12.9-at.% Yb:LGSB nonlinear optical crystal, reporting 0.94 W CW output power at 1070 nm, for a *P_abs_* = 2.8 W at 972 nm, with 44% slope efficiency [29].

In this paper a Nd-doped LGSB crystal is investigated, for the first time to our knowledge, as an efficient laser material, emitting at 1.06 μm. We start by reporting the main crystal growth conditions, structural characterization, and the spectroscopic properties of the Czochralski-grown Nd:LGSB crystal. Then, we present the obtained results concerning the laser emission at 1.06 μm for the case of longitudinally pumping, at 807 nm, with a fiber coupled laser diode. We report on the CW and quasi-CW laser operation characteristics for two 5-at.% Nd:LGSB media, cut along *c-* and *a-* crystallographic axes. Employing the semiconductor saturable absorber mirror (SESAM) technique, we obtained passive Q-switching in a short resonator, as well as passive mode-locking (ML) with a long Z-type resonator. The results are comparable with those reported for a flat-Brewster cut Nd:LSB medium [24]. Our investigations have shown that the Nd:LGSB crystal is a promising candidate as an efficient infrared laser material, with slope efficiencies higher or comparable to those of Nd:YAG or Nd:YVO_4_ crystals in similar experimental conditions [15,16]. Watt-level output power, i.e., 1.35 W at 1.06 μm, is demonstrated in the CW operation, while, with the SESAM-based passive mode-locking technique, ultrashort pulses, 1.43 ps in duration, are achieved without any intra-cavity dispersion compensation mechanism.

## 2. Materials and Methods

### 2.1. Crystal Growth

Good quality 5-at.% Nd:LGSB crystal was grown by the Czochralski technique, in our laboratory, in similar conditions to those of an undoped LGSB crystal reported by Gheorghe et al. [28]. Based on this study, the starting melt composition was selected to be La_0.628_Nd_0.05_Gd_0.572_Sc_2.75_(BO_3_)_4_, corresponding to 5-at.% Nd:LGSB and considering that Nd^3+^ ions substitute the La^3+^ ions. The starting compound was synthesized by the solid-state reaction method. Chemicals including La_2_O_3_, Nd_2_O_3_, Gd_2_O_3_ and Sc_2_O_3_ of 99.999% purity, and B_2_O_3_ of 99.98% purity were used as raw materials. An excess of 5.0-wt.% B_2_O_3_, with respect to the stoichiometric composition, was added to compensate for evaporation losses during the heat treatment. The Nd:LGSB starting compound was charged into Ir crucibles with an inner diameter of 30 mm and a height of 30 mm and melted under N_2_ atmosphere. The growth temperature was in the range of 1480 °C to 1510 °C and the pulling and rotation rates were 2 mm/h and 8 to 10 rpm, respectively. An <0 0 1> oriented single crystal sample of undoped LGSB was used as the seed. The grown crystal was cooled to room temperature with a low cooling rate of 30 °C/h to reduce stress and avoid cracks after growth.

**Figure 1:**
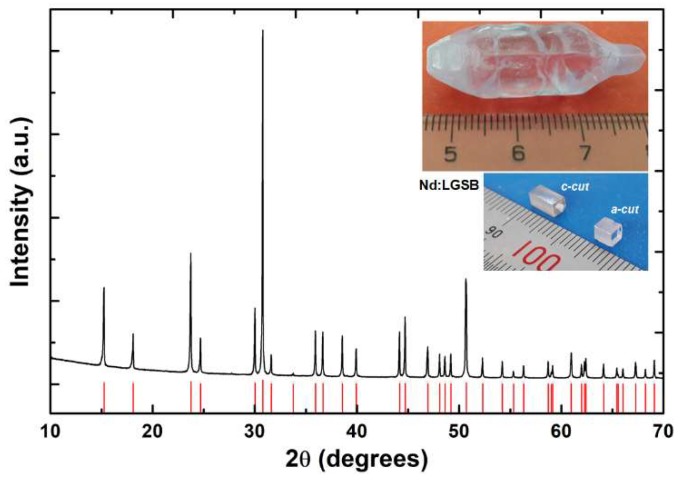
Room temperature XRPD of the Czochralski-grown Nd:LGSB crystal. Red vertical lines correspond to the peaks of *R32* trigonal phase (PDF card 04-018-1225). Insets show the grown boule of Nd:LGSB (top) and the Nd:LGSB active elements used in the experiments (bottom).

Figure 1 shows the X-ray powder diffraction (XRPD) spectrum of the Nd:LGSB grown crystal, together with the Powder Diffraction File (PDF) card 04-018-1225 relative to the trigonal huntite-type phase of LGSB crystal (space group *R32*, Z = 3). From the as-grown crystal, two Nd:LGSB active media of good optical quality were cut along *c*- and *a*- crystallographic axes and their end faces were polished at laser grade. The inset of Figure 1 shows a photo of the grown crystal and the *c*-cut and *a*-cut active elements used in the following investigations.

The X-ray spectrum analysis shows that all the diffraction peaks of the grown crystal are in agreement with those of the undoped LGSB crystal and no additional peaks were found. The unit cell parameters were determinate to be a = 9.7965(6) Å and c = 7.959(1) Å. Taking into account that the segregation coefficient of Nd in LSB crystal is equal to unit, within the uncertainty of the measurement [23], we can assume that the Nd concentration in LGSB grown crystal is about 5.0-at.%.

### 2.2. Spectroscopy

The polarized absorption and emission spectra were recorded at 300 K with a setup consisting of a Jarell Ash and Horiba Jobin-Yvone monochromators (Horiba, Northampton, UK), S20 and S1 photomultipliers, a Ge photodiode, and a lock-in amplifier on line with a computer. The emission spectra were obtained under ~800 nm excitation with a Xe lamp, and the emission cross sections were determined for both (σ and π) polarizations using the Fuchtbauer–Ladenburg method [30].

The lifetime measurement was performed at room temperature by exciting the Nd:LGSB sample at 808 nm with an OPOTEK RADIANT 355 LD optical parametric oscillator laser (Opotek, CA, USA). The decay signals were displayed on a Tektronix 2024B oscilloscope (Tektronics Inc., Beaverton, OR, USA).

### 2.3. Laser Experiments

#### 2.3.1. Free Running Regime

**Figure 2:**
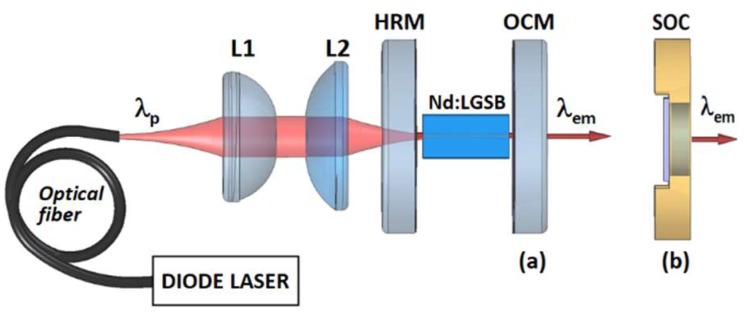
The laser emission experimental set-up. L1, L2: lenses; HRM: high-reflectivity mirror; (**a**) OCM: out-coupling mirror for CW mode operation; (**b**) SOC: saturable absorber output coupler used for passive Q-switching experiments, replacing the OCM.

The set-up used for evaluation of the laser emission performances is shown in Figure 2. In the experiments, two uncoated Nd:LGSB samples, the first cut along the *c-*axis (optically polished planes perpendicular to the *c*-axis) with a length of 6.1 mm and the second cut along *a*-axis (optically polished planes perpendicular to the *a*-axis) with a length of 3.0 mm, were used. The pump was made at 807 nm wavelength (λ_p_) with a fiber-coupled diode laser (LIMO GmbH, Dortmund, Germany) that was operated in quasi-CW mode (pump pulse duration of 1 ms at 10-Hz repetition rate), as well as in CW regime. The fiber end (100-μm diameter, numerical aperture NA = 0.22) was imaged into the Nd:LGSB crystals with a pair of achromatic doublet lenses, L1 and L2. In order to find the pump-beam conditions for optimum laser emission, the pump beam was focused to three different diameters in each Nd:LGSB crystal, of 510, 330, and 230 μm, respectively.

A short (less than 10 mm length) linear plane-plane resonator was built. The rear high-reflectivity mirror (HRM) was coated HR (reflectivity, *R* > 0.999) at the laser emission wavelength (λ_em_) of 1.06 μm and with high transmission, HT (*T* > 0.998) at λ_p_. Various out-coupling mirrors (OCM) with different transmission *T_OC_* at λ_em_ were used (Figure 2a). The mirrors were set as close as possible to the Nd:LGSB medium. In addition, the investigated Nd:LGSB medium was placed in a copper holder that was maintained at 20 °C by circulating water and a Peltier element was also employed . No optical damage of the Nd:LGSB media was observed during the experiments.

#### 2.3.2. Passive Q Switching

It is well known that by accurate design and control of the parameters of a SESAM device, different operation regimes, such as pure Q-switching, Q-switch mode-locking (QML), and continuous-mode locking (CML) could be obtained [31,32,33,34,35,36]. Herein, several Q-switching experiments with SESAM have been performed on the short *a*-cut Nd:LGSB. 

Furthermore, in order to decrease the thermal effects in the laser medium, the pump was done with a beam of 330 μm diameter. Based on the available equipment, two SESAMs (BATOP GmbH, Jena, Germany), which acted also as out-coupling mirrors, were used for passive Q-switching. The first one, denoted by SOC-1, has an out-coupling transmission of 2.2%, 1.1% modulation depth, 0.7% non-saturable loss, 60 μJ/cm^2^ saturation fluence, and a 1 ps recovery time. The second device, SOC-2, has an increased 3.2% transmission, 1.7% modulation depth, 1.0% non-saturable loss, 90 μJ/cm^2^ saturation fluence, and a 1 ps recovery time. The OCM mirror of the set-up (Figure 2a) was replaced by each SESAM, i.e., SOC device (Figure 2b). Although the SOC device was mounted on a copper holder, during the experiments no additional cooling was necessary and no optical damage was observed.

#### 2.3.3. Passive Mode-Locking

**Figure 3:**
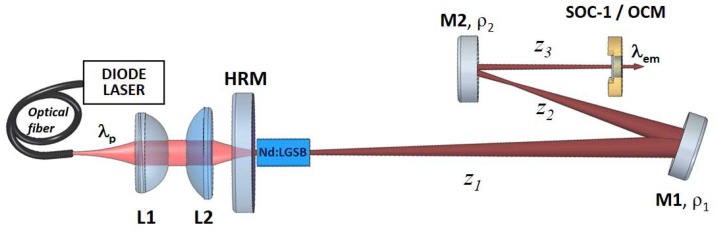
The laser resonator designed for ML experiments; *z*_1_, *z*_2_, *z*_3_, the resonator arm lengths; M1, M2, folding mirrors; ρ_1_, ρ_2_, radius of curvatures.

The uncoated *a*-cut Nd:LGSB crystal was further used in ML experiments, together with the SOC-1 device. For this purpose, a Z-type resonator was built up, as depicted in Figure 3. The pump-beam conditions were those used in the Q-switching experiments. We mentioned that in CW experiments that the stability of the linear plane-plane resonator versus the pump power absorbed in Nd:LGSB was investigated. Next, the focal length of the thermal lens, induced by optical pumping in Nd:LGSB, was evaluated based on the model proposed by Innocenzi et al. [37]. Then, using Paraxia Plus Software for ABCD simulations, the resonator laser mode was downsized inside Nd:LGSB and also on SOC-1, aiming good mode matching together with strong focusing on the SESAM device for enhanced longitudinal mode coupling.

As shown in Figure 3, the resonator contains two plane-concave M1 and M2 mirrors, with radius of curvatures at ρ_1_ = 350 mm and ρ_2_ = 100 mm, respectively. The Nd:LGSB crystal was placed close to the plane HRM mirror. Distance *z*_1_, between HRM and M1, and distance *z*_2_, from M1 to M2, were 730 and 290 mm, respectively. The distance between M2 and the out-coupling device (SOC-1 or a plane OCM) was set to *z*_3_ = 245 mm. According to our modelling, the laser beam radius in Nd:LGSB was kept constant, with a ~150 μm radius, within the resonator stability zone. In addition, the laser beam incident on SOC-1 changed slowly, decreasing from a 280 µm radius, close to the edge of the stability zone, down to a 230 µm radius when the pump power was reduced down to the threshold value. The high value of the laser beam size on SESAM (SOC-1), which can be responsible for a small nonlinearity, is compensated by the small out coupling of the resonator (only 2.2% transmission) and, thus, high intra-cavity power is expected. In this case, too, no optical damage of the Nd:LGSB medium was observed during the experiments.

## 3. Results and Discussions

### 3.1. Spectroscopic Properties

**Figure 4:**
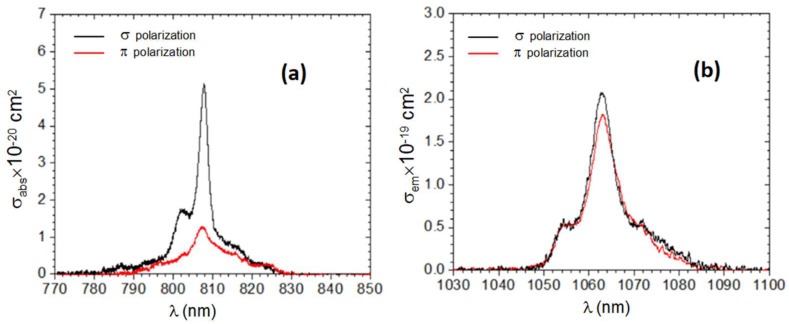
(**a**) Polarization selective absorption cross section of an *a*-cut Nd:LGSB crystal. (**b**) The emission cross section of Nd:LGSB for the ^4^F_3/2_ → ^4^I_11/2_ transition at 1063 nm.

The absorption cross sections (σ_abs_) in the 800 nm range for the *a*-cut Nd:LGSB crystal are presented in Figure 4a. In the case of σ polarization, the peak absorption of ~5.1 × 10^−20^ cm^2^ is observed at 807.8 nm, with a spectrum bandwidth of 3.2 nm. For the *π* polarization, the absorption spectrum is centered at 807.1 nm with a maximum of 1.3 × 10^−20^ cm^2^ (i.e., four times lower than for the σ polarization), but with a 2.6-times larger 8.3-nm bandwidth. Figure 4b illustrates the emission cross sections (σ_em_) corresponding to a ^4^F_3/2_ → ^4^I_11/2_ transition, for both σ and π polarizations. The emission cross section at 1063 nm was 2.1 × 10^−19^ cm^2^ for σ polarization and 1.8 × 10^−19^ cm^2^ for π polarization. These values are comparable to that of Nd:YAG (~3.3 × 10^−19^ cm^2^ [23]). The emission bandwidth (FWHM) of Nd:LGSB was determined to be 6.5 nm and 7.3 nm for σ and π polarization, respectively. These values are much larger than that of Nd:YAG (~0.5 nm), indicating that Nd:LGSB could be used in designing ultrashort pulsed laser systems operating at few ps up to hundreds of fs duration.

**Figure 5:**
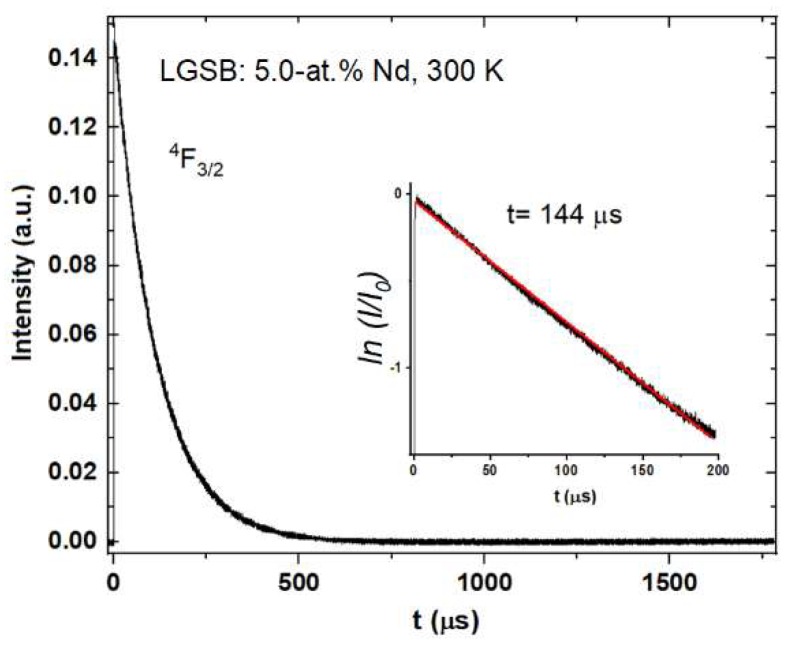
^4^F_3/2_ decay curve measured at room temperature. Inset shows a logarithmic plot of the intensity and its corresponding linear fit (red line).

Figure 5 shows the decay curve of the ^4^F_3/2_ metastable level. By fitting the decay curve, a value of 144 μs was determined for the fluorescence lifetime (*τ_f_*). It is worth mentioning that a close value (of 150 μs) was reported for a Nd:LSB crystal [38] with a similar Nd doping concentration.

Table 1 offers an overview regarding the key spectroscopic parameters of various Nd-doped active crystals as compared to Nd:LGSB crystal. It can be observed that 5.0-at.% Nd:LGSB crystal presents 2.2 times higher σ_abs_ than Nd:GdCOB and Nd:YAB crystals in π polarization, while for σ polarization, σ_abs_ is almost half of those reported for Nd:GdCOB or Nd:YAB crystals. Also, it can be observed that the saturation intensity of the Nd:LGSB crystal is on the same order of magnitude with those of Nd:LSB and Nd:YAB laser crystals. Compared to Nd:YAB, the lifetime of the ^4^F_3/2_ metastable level of Nd:LGSB crystal is 2.4 times higher, which is a good indicator for laser operation in a high peak power pulsed regime. Moreover, even at a higher concentration of Nd^3+^ ions in LGSB (5.0-at.%) than in the YVO_4_ matrix (1.0-at.%), the lifetime of the metastable level is still longer (144 µs compared to 95 µs). From another point of view, when ultrashort pulses are desired, the Nd:LGSB crystal presents the advantage of having an emission bandwidth sufficiently large to support pulses of sub ps duration, compared to the other crystals from Table 1. In comparison with well-known Nd:YAG and Nd:YVO_4_ crystals, the Nd:LGSB crystal has emission bandwidths (6.5 nm in σ polarization and 7.3 nm in π polarization) of about ten times higher than those reported for Nd:YAG and Nd:YVO_4_ crystals. Based on all these data, we can conclude that the Nd:LGSB crystal could compete well with previously developed Nd-doped laser crystals in various applications which need ultrashort high peak power pulses.

### 3.2. Laser Emission

#### 3.2.1. Free Running Regime

**Figure 6:**
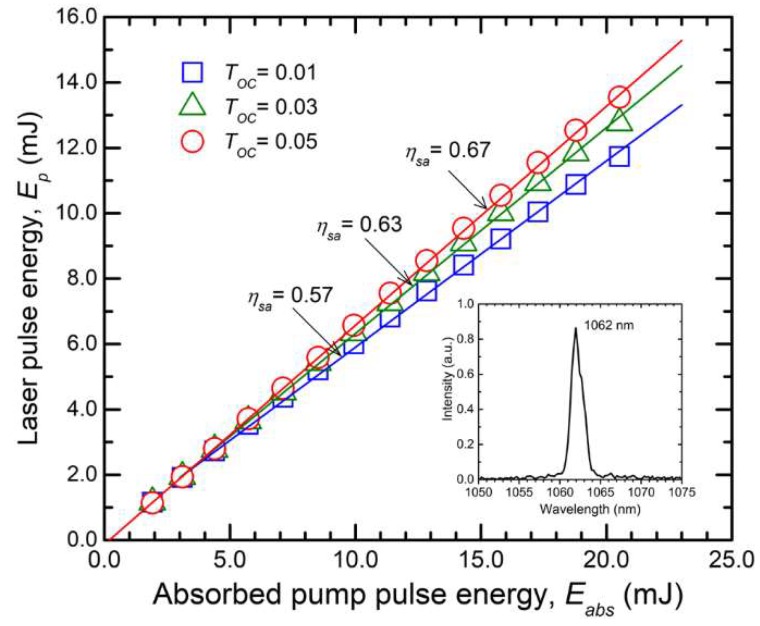
Laser pulse energy, *E*_p_, yielded by the *c*-cut Nd:LGSB crystal versus absorbed pump-pulse energy, *E*_abs_, for a pump-beam diameter of 230 µm. Inset shows the laser emission spectrum.

Figure 6 presents the results obtained with the *c*-cut Nd:LGSB medium, for the case of pump beam diameter of 230 µm and under the quasi-CW mode of operation. With an OCM of *T_OC_* = 0.01 the laser yielded pulses with *E_p_* = 11.7 mJ for an absorbed pump pulse energy, *E_abs_* = 20.5 mJ. The pump beam absorption efficiency was η*_a_* = 0.99. The slope efficiency (with respect to *E_abs_*) was η*_sa_* = 0.57. On the other hand, when an OCM with *T_OC_* = 0.05 was used, the pulse energy increased at *E_p_* = 13.5 mJ (*E_abs_* = 20.5 mJ), corresponding to an overall optical-to-optical efficiency (with respect to *E_abs_*) of η*_oa_* = 0.66. The slope efficiency improved to η*_sa_* = 0.67. Furthermore, the *c*-cut Nd:LGSB crystal emitted pulses with *E_p_* = 12.1 mJ (at η*_oa_* = 0.64) when the pump was made with the 330 µm diameter beam and the resonator was equipped with an OCM of *T_OC_* = 0.05; slope efficiency was η*_sa_* = 0.66. Increasing the pump-beam diameter at 510 µm decreases *E_p_* at 11.2 mJ (at η*_oa_* = 0.55) and reduces slope efficiency down to η*_sa_* = 0.57.

In the case of the *a*-cut Nd:LGSB crystal, the highest performances were also obtained under the pump with the 230 µm diameter beam. For the OCM with *T_OC_* = 0.05, laser pulses with *E_p_* = 9.5 mJ at *E_abs_* = 15.3 mJ (η*_oa_* = 0.62) were emitted; the slope efficiency amounted to η*_sa_* = 0.65. The pump-beam absorption efficiency for this crystal was η*_a_* = 0.74. A Findlay–Clay analysis [41] of the thresholds of emission concluded that the resonator residual losses, *L_i_*, were around 0.01 for both Nd:LGSB media, proving the good quality of the laser crystals. In addition, the laser emission spectrum was centered at 1062 nm, being 1.7 nm wide (inset of Figure 6).

**Figure 7:**
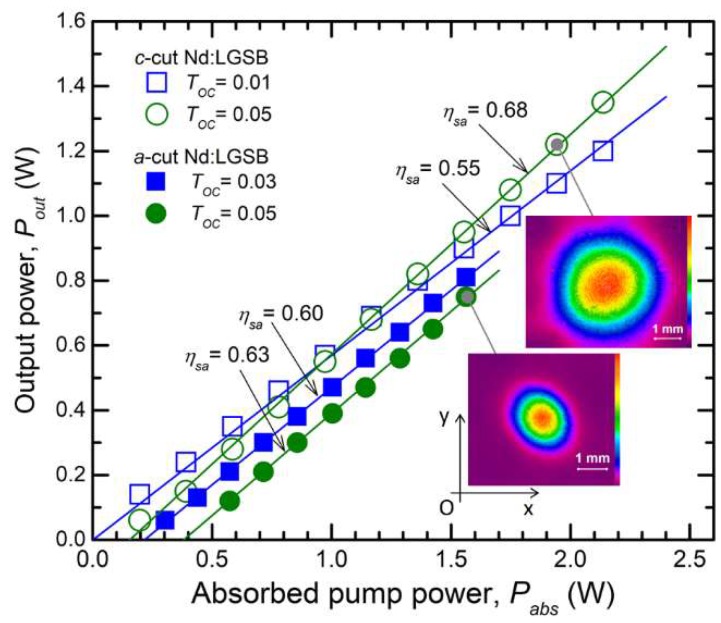
Comparative CW laser operation for *c*-cut and *a*-cut Nd:LGSB. The near field distributions are shown for the indicated points.

Next, the CW laser performances for both Nd:LGSB crystals are illustrated in Figure 7. With an OCM of *T_OC_* = 0.05, the *c*-cut Nd:LGSB medium yielded maximum output power of *P_out_* = 1.35 W at an absorbed pump power of *P_abs_* = 2.14 W; thus, the optical-to-optical efficiency was η*_oa_* = 0.63. The slope efficiency amounted to *η_sa_* = 0.68. In the case of the *a*-cut Nd:LGSB crystal, the highest *P_out_* = 0.81 W was measured with an OCM with *T_OC_* = 0.03; the crystal absorbed *P_abs_* = 1.55 W (η*_oa_* = 0.52). The slope efficiency was η*_sa_* = 0.60. On the other hand, the slope efficiency improved to η*_sa_* = 0.63 for an OCM with *T_OC_* = 0.05, but less power, *P_out_* = 0.75 W, was obtained at *P_abs_* = 1.55 W. A Glan–Taylor calcite polarizer (with extinction ratio higher than 100.000:1) was used to determine the laser beam polarization. It was concluded that the *c*-cut Nd:LGSB yielded a randomly-polarized beam, whereas the beam delivered by the *a*-cut Nd:LGSB crystal was linearly polarized (100:1 polarization ratio). The medium outputted σ polarized laser beam, which corresponds to the polarization with highest emission cross section (as shown in Figure 4b). The near field distribution (inset of Figure 7) was circular in the case of the *c*-cut crystal, but shows a slightly elliptical shape for the *a*-cut medium. This could be caused by some temperature gradients induced by non-uniform cooling, or may result from different thermal coefficients along the two axes of the Nd:LGSB uniaxial crystal. We mention that the beam near-field distributions were recorded with a Spiricon camera (model SP620U, 190- 1100 nm spectral range) that was positioned 300 mm far away from OCM. In this condition, the CW laser beam delivered by the *c*-cut Nd:LGSB had a diameter (O*x* × O*y* axis) of 4.8 mm × 4.9 mm (1/e^2^ definition), while the *a*-cut Nd:LGSB outputted a laser beam 2.2 mm × 2.3 mm in diameter.

#### 3.2.2. Passive Q-switching

**Figure 8:**
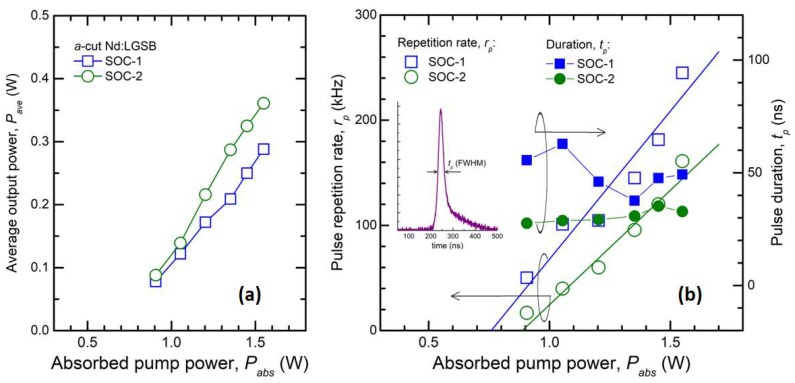
(**a**) Average output power of Nd:LGSB laser passively Q-switched by SESAM (SOC-1, SOC-2); (**b**) Pulse repetition rate and pulse duration. Inset shows a typical laser pulse shape.

The average output power, *P_ave_*, obtained by Q-switching with SESAM of the *a*-cut Nd:LGSB laser is shown in Figure 8a. The maximum *P_ave_* = 0.29 W was measured with SOC-1. At this pump level (*P_abs_* = 1.55 W) the laser runs at high ~261 kHz repetition rate, *r_p_*, and delivers pulses with a duration of *t_p_* = 49.2 ns (Figure 8b). Consequently, the Q-switch laser pulse energy and the pulse peak power were determined as 1.1 μJ and 22.4 W, respectively. We mention that the pulse duration was measured with a fast 35 ps rise time photodiode (ALPHALAS GmbH, Germany), connected to a high 2.5 GHz bandwidth oscilloscope (Tektronix DPO7254). The power increased at *P_ave_* = 0.36 W when SOC-2 was employed for the Q-switch regime. The laser maximum repetition rate was *r_p_* = 161 kHz (thus, lower than that observed with SOC-1), whereas the laser pulse duration was shorter, *t_p_* = 32.8 ns. The inset of Figure 8b presents such a pulse shape. For this saturable absorber, the pulse energy increased at 2.2 μJ and the pulse peak power reached ~67 W. One could observe (Figure 8b) that with SOC-2 the repetition rate, *r_p_*, varied linearly with the absorbed power, *P_abs_*, whereas the pulse duration, *t_p_*, was almost constant on the entire pump power range. On the other hand, high fluctuations were observed for *r_p_* in the case of SOC-1. Furthermore, the pulse duration was long, *t_p_* = 55.6 ns at low *P_abs_* = 0.9 W, and shortened to 49.2 ns for *P_abs_* = 1.55 W. One reason for this behavior could be the thermal effects in Nd:LGSB, which are enhanced by low power extraction from the medium, that could vary the laser beam caustic inside the laser resonator.

#### 3.2.3. Passive Mode-locking

**Figure 9:**
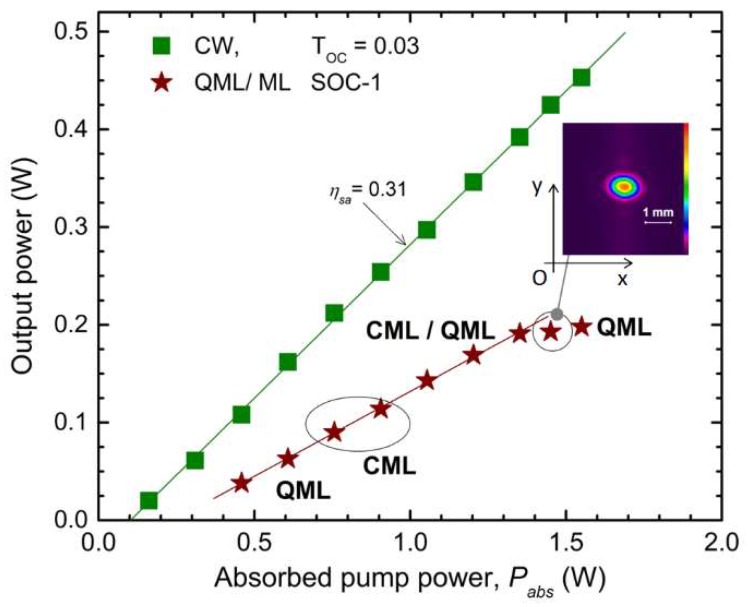
Output power versus P_abs_ for CW and QML/CML Nd:LGSB laser. Inset shows the 2D beam profile for the CML operation at the indicated point.

The Z-type laser resonator was designed to operate within the stability zone up to the maximum pump power used in the experiments, *P_abs_*~1.5 W. At this point, the Nd:LGSB thermal lens was evaluated to have a ~65 mm focal length. The performances of laser emission yielded by the *a*-cut Nd:LGSB in the Z-type resonator are shown in Figure 9. First, CW operation with an output power of *P_out_* = 0.45 W at *P_abs_* = 1.55 W (η*_oa_* = 0.29) was obtained with a plane OCM having *T_OC_* = 0.03 (this mirror was used instead of SOC-1, Figure 3). The slope efficiency was η*_sa_* = 0.31. The pump power at the lasing threshold was as low as *P_abs_* = 0.1 W.

QML and CML regimes were observed when the OCM was replaced with SOC-1, the SESAM device. The laser began to lase at *P_abs_* = 0.24 and operated in QML mode up to *P_abs_* = 0.61 W, where the output power was *P_ave_* = 0.06 W. Next, CML was recorded on a narrow window, *P_abs_* up to ~0.91 W (*P_ave_* = 0.11 W), followed by a wide range, *P_abs_* up to ~1.35 W, within which mixed CML and QML operation was noticed. Next, CML with output power *P_ave_* = 0.19 W was recorded for *P_abs_* = 1.45 W. The inset of Figure 9 represents the distribution of the CML laser beam, for which the M^2^ factor was measured to be 1.23. The beam diameter, which was determined in conditions similar to those used for CW operation, was 1.5 mm × 1.1 mm. It was observed that the beam delivered by the ML Nd:LGSB laser is elliptical, with the stronger emission along the *a*-axis, which corresponds to the polarization with the higher emission cross-section. However, such ellipticity is expected, due to the nonzero folding angles of the laser cavity. This issue could be managed in future experiments with an improved resonator design that would allow selecting the laser mode with the desired dimensions. Finally, for the maximum pump power, *P_abs_* = 1.55 W, the laser operated in the QML regime with *P_ave_ =* 0.20 W. At this point, the laser ran at a 72 kHz repetition rate; thus, the pulse energy was estimated to be 2.75 µJ. The pulse duration was 7.3 µs (FWHM), indicating a low ~0.38 W pulse peak power. A further increase of the pump power drastically reduced the output, indicating that the resonator had reached the edge of the stability zone (in accordance with the resonator design). The laser crystal was uncoated and it was also not wedged [36]. The etalon effects could, therefore, be responsible for the mixed CML/QML behavior. Power scaling of the laser is then important for reaching the critical energy beyond which the Q-switch instabilities will be totally suppressed.

**Figure 10:**
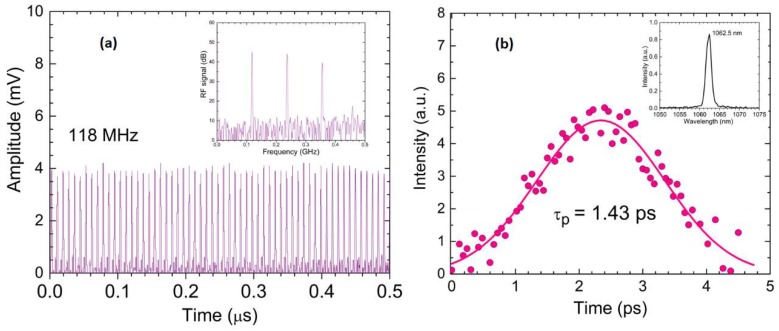
(**a**) CML pulses recorded at 50 ns time scale. Inset shows the corresponding RF signal. (**b**) Autocorrelation trace measured at *P_abs_* = 1.45 W. Inset is the 1.06 µm emission spectrum of QML/CML Nd:LGSB laser.

A typical CML pulse train at *P_ave_* = 0.19 W is shown in Figure 10a. The repetition rate is 118 MHz, which fits very well with the expected value for the 1.27 m long resonator used in the experiments. The average power was measured at each minute within a 45 min operation range, showing fluctuations of ~0.11% (RMS deviation). In addition, the radio frequency spectrum was determined with a Fourier transformation applied to the oscilloscope trace, recorded at a 50 ns time scale. A 45 dB maximum separation of the signal from noise was achieved, as presented in inset of Figure 10a. Figure 10b illustrates the autocorrelation trace, at the same pump level. The intensity of the autocorrelation signal was recorded with the oscilloscope at every 0.06 ps time delay between the pulses. The autocorrelation function of the ML pulse was constructed with a Delta Single-Shot Intensity Autocorrelator (Minioptic Technology). As shown in Figure 10b, a value of 1.43 ps was obtained for the ML pulse, with a Gaussian distribution approach. The corresponding limited value for the ~1.70 nm bandwidth is 0.98 ps. The laser emission spectrum is shown in the inset of Figure 10b for the QML/ML Nd:LGSB laser, at *P_abs_* = 1.45 W. As compared to the spectrum recorded for quasi-CW pumped Nd:LGSB, in this case, the *a*-cut Nd:LGSB medium, operating in the QML/CML regime, presented similar bandwidth, while the emission spectrum peaked to 1062.45 nm. The achieved pulse duration was 1.46 times the transform-limited value, indicating that Nd:LGSB crystal is a promising material for generation of ultrashort pulses with few ps pulse durations and a close to sub-ps temporal range. Pulse energy and the corresponding peak power of the CML Nd:LGSB laser were 1.63 nJ and 1.14 kW, respectively.

The results obtained here are comparable with those reported by B. Braun et al in the case of a Nd:LSB passive ML with an antiresonant Fabry–Perot saturable absorber [24]. They achieved a pulse duration of 2.8 ps at 1062 nm and *P_ave_* = 400 mW at 1.2 W pump power, under diode pumping. In addition, with Ti:sapphire pumping, they obtained 1.6 ps at a 177 MHz repetition rate. The spectral bandwidth of the Nd:LSB ML laser was 1.1 nm, thus shorter than that reported in this work with the *a*-cut Nd:LGSB crystal. The 1.46 times transform limited value is somehow expected with this ML laser set-up, where the gain medium is situated at one end of the resonator and the saturable absorber at the opposite one, due to strong spatial hole burning effects [31]. Better results could be anticipated once dispersion compensation mechanisms like prism pairs are inserted in the long cavity. Additionally, a wedged Nd:LGSB crystal may lead to reduced etalon effects and stronger selectivity of the oscillating modes, thus outputting a stable ML operation with shorter transform limited pulses. In this respect, further mode locking experiments on Nd:LGSB crystal samples with one or two wedged facets, analogous to our previous studies on wedge-cut Nd:YVO_4_ crystals [36], will be performed.

## 4. Conclusions

In summary, in this work, we report for the first time, to our knowledge, on laser emission performances at 1.06 µm, obtained from a Nd:LGSB crystal grown by the Czochralski method. In quasi-CW operation, a *c*-cut, 6.1-mm long, and 5.0-at.% Nd:LGSB uncoated crystal yielded pulses with 13.5 mJ energy at 0.66 overall optical-to-optical efficiency. The laser operated with 0.67 slope efficiency. Furthermore, 1.35 W CW output power at 0.63 optical-to-optical efficiency was obtained from the same crystal and a high 0.68 slope efficiency was determined. Laser performances of an *a*-cut, 3-mm long, and 5.0-at.% Nd:LGSB uncoated crystal were measured as well. Furthermore, a passive Q-switch mode of operation was obtained from the *a*-cut Nd:LGSB crystal using SESAM output couplers. Pulses with 2.2 µJ energy and 32.8 ns in duration at a 161 kHz repetition rate (0.36 W average power) were achieved. Additionally, QML and CML regimes were investigated in a long Z-type resonator. A CML regime with 0.19 W average power, 1.63 nJ pulse energy, and 1.43 ps pulse duration was obtained. Future ML experiments with dispersion compensation within the cavity could shorten the pulse duration significantly. These preliminary results suggest that a Nd:LGSB single crystal, which can be grown to a large size and with a good optical quality by the Czochralski method, is quite promising for the development of efficient and compact 1 µm laser sources operating under various regimes. Improvements of the Nd:LGSB infrared laser emission are expected to be achieved by AR-coating of the laser medium, optimization of the Nd concentration and crystal length, and, in the case of ML experiments, by using dispersion compensation prisms inside the cavity and Nd:LGSB media with wedge-cut facets. Having the benefit of large emission bandwidth, needed for ultrashort pulses with high peak power, together with the possibility of obtaining efficient self-frequency doubling (SFD), Nd:LGSB crystals could be used for developing a compact visible laser source with different applications, such as high resolution microscopy, coherent anti-Stokes Raman spectroscopy (CARS), and so on. Moreover, for applications which require a high degree of compactness and for low cost visible laser systems, Nd:LGSB crystals could be employed in an SFD configuration, instead of the conventional microchip visible lasers, based on frequency doubling of Nd:YVO_4_ or Nd:YAG infrared laser emission in various nonlinear optical crystals. Such aspects will be considered in future work.

## Figures and Tables

**Table 1:** Spectroscopic properties comparison for various Nd-doped laser crystals.

Material	σ_abs_ (10^−20^ cm^2^)	τ*_f_* (µs)	λ_em_ (nm)	Emission Bandwidth (nm)	σ_em_ (10^−20^ cm^2^)	Saturation Intensity (kW/cm^2^)
Nd:LGSB (this work)	5.1 (π) 1.3 (σ)	144 (5.0-at.% Nd)	1063	6.5 *(*σ) 7.3 (π)	2.1 (σ) 1.8 (π)	6.18
Nd:YAG [23]	7	240	1064	0.5	3.3	2.36
Nd:YVO_4_ [13]	27	95 (1.0-at.% Nd)	1064	0.8	19.5	1
Nd:LSB [23,38]	7.1	118 (10-at.% Nd) 150 (5.0-at.% Nd)	1063	4	1.3 [23] 1.45 [38]	12.2 [23] 8.6 [38]
Nd:GdCOB [39]	2.3	98 (4.0-at.% Nd)	1061	3.5	0.29	65
Nd:YAB [40]	2.3	60 (4.0-at.% Nd)	1061	9	10	3.12
